# Quantifying Arctic lower stratospheric ozone sources in winter and spring

**DOI:** 10.1038/s41598-018-27045-5

**Published:** 2018-06-12

**Authors:** Chen Pan, Bin Zhu, Jinhui Gao, Xuewei Hou, Hanqing Kang, Dongdong Wang

**Affiliations:** 1 Key Laboratory for Aerosol-Cloud-Precipitation of China Meteorological Administration, Nanjing University of Information Science & Technology, Nanjing, China; 2 Collaborative Innovation Center on Forecast and Evaluation of Meteorological Disasters, Nanjing University of Information Science & Technology, Nanjing, China; 3 Key Laboratory of Meteorological Disaster, Ministry of Education (KLME), Nanjing University of Information Science & Technology, Nanjing, China; 4 Joint International Research Laboratory of Climate and Environment Change (ILCEC), Nanjing University of Information Science & Technology, Nanjing, China

## Abstract

The dynamical and chemical characteristics of unusually low Arctic ozone events in 2005 and 2011 have been well-studied. However, the quantitative identification of Arctic ozone sources is lacking. Here, we use tagged ozone tracers in a numerical simulation to quantify the contributions to Arctic lower stratospheric ozone (ARCLS_O_3_) at diverse latitudes in winter and spring from 2005–2011. We demonstrate that the northern mid-latitudinal stratosphere steadily contributes approximately half of ARCLS_O_3_. The absolute contributions during February have evident variations, which are smaller in cold years (151.3 ± 7.0 Dobson units (DU) in 2005 and 139.0 ± 7.4 DU in 2011) and greater in warm years (182.6 ± 7.3 DU in 2006 and 164.6 ± 7.4 DU in 2009). The tropical stratosphere is also an important source. During February, its absolute contributions are 66.5 ± 11.5 DU (2005), 73.1 ± 4.7 DU (2011), 146.0 ± 9.0 DU (2006), and 153.7 ± 7.0 DU (2009). Before and after stratospheric warming, variations in the tropical components of ARCLS_O_3_ (51.8 DU in 2006 and 77.0 DU in 2009) are significantly larger than those in the mid-latitudinal components (17.6 DU in 2006 and 18.1 DU in 2009). These results imply that although the mid-latitudinal components of ARCLS_O_3_ are larger, the tropical components control stratospheric temperature-induced ARCLS_O_3_ anomalies in winter and spring.

## Introduction

The variability of ozone (O_3_) loss in the Arctic has always been much greater than that in the Antarctic^1,2^. The main driver of the different chemical ozone loss levels in the Arctic and the Antarctic is the difference in temperature^3^. The year 2005 and 2011 exhibit two cold winters in the Arctic stratosphere, where ozone losses in the Arctic lower stratosphere are particularly intense compared to those in most historical years^4,5^. In addition to the two cold Arctic winters, unusually strong and prolonged (nearly a month long) sudden stratospheric warmings occurred in January 2006 and January 2009, with higher Arctic ozone being observed during winter and spring^6,7^.

The lifetime of stratospheric ozone varies substantially with altitude (e.g., it ranges from ~140 days at 20 km to ~12 days at 40 km^8^). In addition, the lifetime of stratospheric ozone is also latitudinally and seasonally dependent. For instance, its lifetime in the lower stratosphere is very long during polar nights. Tropical ozone production and the large-scale transport of ozone (below a 30-km height), such as the Brewer-Dobson circulation, are generally thought to dominate the distribution of ozone in the lower stratoshpere^9^, especially during winter^10^. The quantitative source apportionment of stratospheric ozone can provide further insight or information on stratospheric ozone. However, in nature, the origin of ozone is difficult to quantify via measurements. The tagged ozone tracer method, in which ozone tracers are “tagged” at their chemically-produced geographical source region and forced to undergo the same processes in a used model as those of regular ozone, is an effective tool to quantitatively identify the contribution of ozone from one region to another. However, this method is primarily used to study the sources of tropospheric ozone^11,12^ currently. As far as we know, Grewe^9^ applied this method to study the sources of stratospheric ozone, which separated ozone contributions from tropical and extra-tropical regions and suggested that the tropics were not a dominant source region of ozone at high-latitudes. However, the contributions from mid- and high-latitudes were not separated in his study. Currently, the contributions from various latitudinal zones to the Arctic stratospheric ozone are still unquantified, and a relevant study has not been performed.

Characterized by extremely cold and warm winters, the large interannual variability of Arctic stratospheric winters is an interesting phenomenon in climate science^6^. The thermal and dynamic conditions in Arctic winters are related to planetary wave activities. Weaker wave activities lead to very low polar stratospheric air temperatures, while intense wave forcing causes warm polar stratospheric air temperatures^13,14^, which impact stratospheric ozone loss and recovery. Extremely cold winters (2005 and 2011) and warm winters (2006 and 2009) in the Arctic occurred frequently from 2005–2011. Though these unique Arctic winters have been widely studied, whether there are differences in the contributions to Arctic stratospheric ozone from various latitudinal zones between cold and warm winters is an interesting question that has been previously overlooked. In addition, satellite data, i.e., Earth Observing System Microwave Limb Sounder (MLS), have provided the daily vertical measurements of various stratospheric chemical constituents since August 2004^15^, which can facilitate the assessment of the performance of the Whole Atmosphere Community Climate Model (WACCM)^16^ when simulating ozone and related species. Therefore, these Arctic winters provide good cases for studying the budget of polar ozone in different extreme years. In this study, we use the tagged ozone tracer method to quantify the contributions from various latitudinal zones to lower stratospheric ozone in the Arctic in winter and spring from 2005–2011 and present the initial comparisons between the sources of Arctic ozone during cold winters (2005 and 2011) and warm winters (2006 and 2009). This work could improve our understanding of the sources and budget of Arctic stratospheric ozone in winter and spring.

In this study, the evaluation of simulated ozone and the source apportionment of lower stratospheric ozone in the Arctic are provided in the Results section. A summary is presented in the Conclusions section. Finally, descriptions of the MLS data, the WACCM model, and the tagged ozone tracer method are provided in the Methods section.

## Results

### Evaluation of simulated ozone

**Figure 1:**
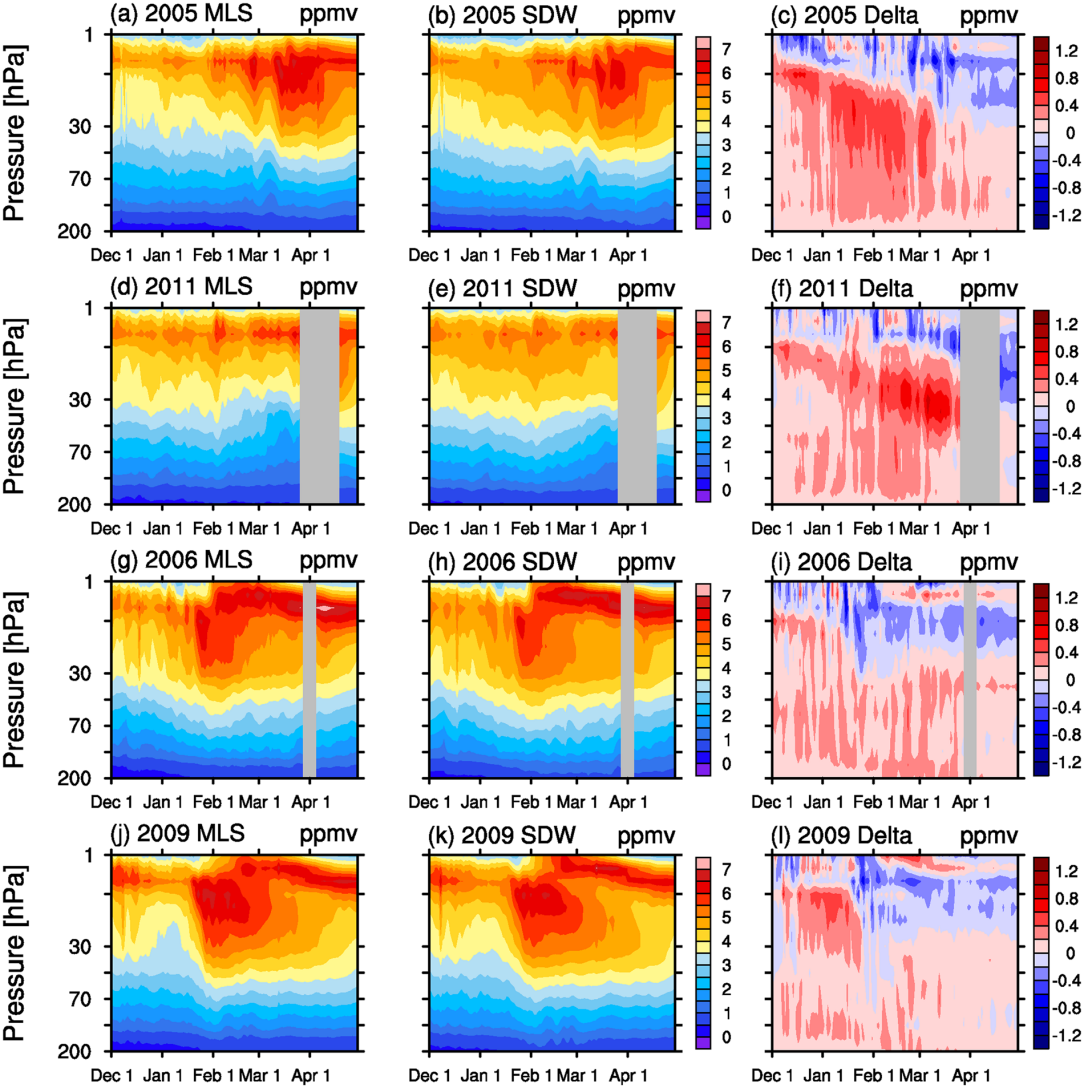
Evolutions of daily (left) MLS observations, (center) SD-WACCM simulations, and (right) their differences in O_3_ (unit: ppmv) over the Arctic from December to April of (first row) 2005, (second row) 2011, (third row) 2006, and (fourth row) 2009. The grey areas indicate where data were either missed or acquired outside of the Arctic.

**Figure 2:**
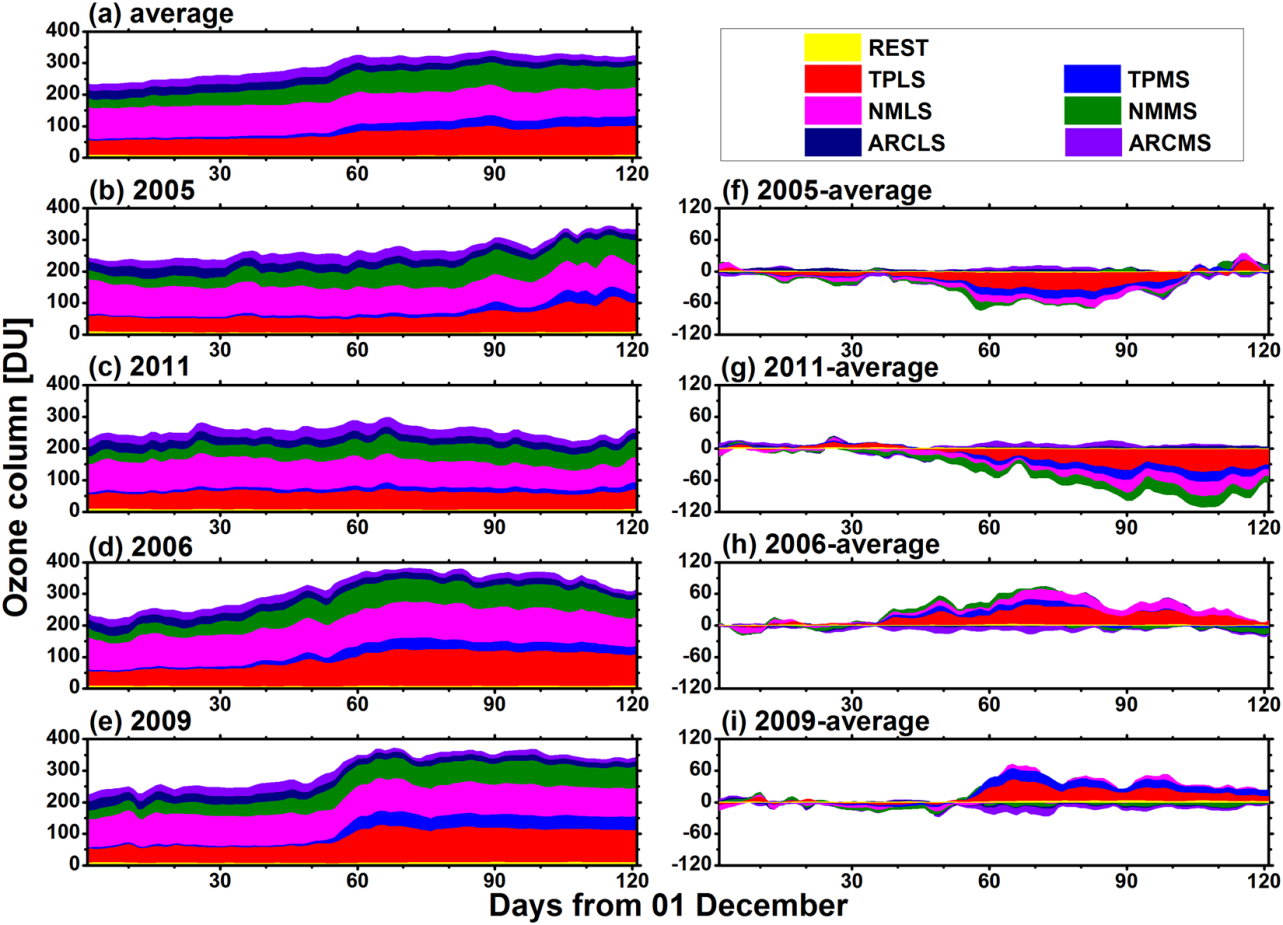
The mean contributions from seven ozone source regions to the lower stratospheric ozone column (unit: DU) over the Arctic region from December to March: (**a**) the 7-year average from 2005–2011, (**b**) 2005, (**c**) 2011, (**d**) 2006, and (**e**) 2009. All values are vertically integrated results from the tropopause to 20 hPa. (**f**–**i**) Show the differences between the ozone contributions in the four specific years (2005, 2011, 2006, and 2009) and the 7-year average, respectively. The abbreviations for the source regions have been explained in Table 1.

Because the mean total column of ozone in March north of 63°N has been regularly used to investigate the development of polar ozone^1,17^, the Arctic was defined as areas north of 63°N in this study. Comparisons between the MLS measurements and the specified dynamics version of WACCM (SD-WACCM)^18,19^ simulated Arctic ozone between 200 and 1 hPa in cold (2005 and 2011) and warm (2006 and 2009) years are shown in Fig. 1. SD-WACCM was sampled at the MLS observation positions with latitudinal and longitudinal accuracies of ±0.5°. The overall distribution and evolution of the SD-WACCM O_3_ were similar to those via the MLS measurements for these four years. The differences between SD-WACCM and MLS O_3_ during these four years are illustrated in the rightmost column of Fig. 1. The precision of the volume mixing ratio (VMR) of O_3_ in the individual MLS profiles was 0.02–0.2 ppmv between 215 and 1 hPa^15^. Below 10 hPa, the SD-WACCM O_3_ was generally 0.2–0.4 ppmv greater than the MLS O_3_ in 2005, 2006, and 2009, indicating an overestimation of SD-WACCM O_3_. Moreover, SD-WACCM also generally overestimated O_3_ by 0.2–0.6 ppmv in 2011. Supplementary Figs S1–S4 show the comparisons between the MLS observed and SD-WACCM simulated temperatures, nitrous oxide (N_2_O), chlorine monoxide (ClO), and hydrochloric acid (HCl) across the Arctic in 2005, 2011, 2006, and 2009, respectively. Qualitatively, SD-WACCM can characterize the evolutions of these measured species or variables reasonably well. However, the simulated temperatures were generally 0.6–3 K higher than the MLS temperatures between 100 and 1 hPa each year. For SD-WACCM, because heterogeneous chemistry is temperature-dependent, the model generally overestimated HCl and underestimated ClO in the lower stratosphere during winter, implying insufficient chlorine activation. These results were consistent with the overestimation of O_3_ in Fig. 1. Similar results have also been achieved in previous studies^20,21^. Brakebusch *et al*.^20^ suggested that SD-WACCM overestimated ozone and HCl in the lower stratosphere inside the Arctic vortex during the Arctic winter of 2005 (see the Fig. 2 in their study). Solomon *et al*.^21^ noted that SD-WACCM overestimated the total column of ozone at 82°N from late February to March and overestimated the concentration of HCl at 82°N and 53 hPa in January 2011 (see Figs 2, 4 in their study). In general, SD-WACCM reasonably reproduced the observed seasonal variations in Arctic stratospheric ozone.

### Sources of Arctic lower stratospheric ozone

In this study, the stratosphere over the tropics and the Northern Hemisphere was divided into six source regions (Table 1) to separate the contributions from low-, mid-, and high-latitudinal zones to Arctic lower stratospheric ozone. The rest of the atmosphere was regarded as the seventh region and is denoted as REST. The region of interest was the Arctic lower stratosphere (i.e., altitudes ranging from the tropopause to 20 hPa). Figure 2a shows the mean contributions from seven source regions to the column of Arctic lower stratospheric ozone from December to March from 2005–2011. The corresponding results in 2005, 2011, 2006, and 2009 are shown in Fig. 2b–e, respectively. Meanwhile, the differences between the results during the four specific years and the 7-year average results are shown in Fig. 2f–i, respectively.

**Table 1:** The six ozone source regions defined in this study.

Region	Latitude	Pressure
The tropical lower stratosphere (TPLS)	30°S–30°N	TPP–20 hPa
The tropical middle and upper stratosphere (TPMS)	30°S–30°N	≤20 hPa
The northern mid-latitudinal lower stratosphere (NMLS)	30°–63°N	TPP–20 hPa
The northern mid-latitudinal middle and upper stratosphere (NMMS)	30°–63°N	≤20 hPa
The Arctic lower stratosphere (ARCLS)	63°–90°N	TPP–20 hPa
The Arctic middle and upper stratosphere (ARCMS)	63°–90°N	≤20 hPa

TPP is an abbreviation for pressure at the tropopause, which is dynamically calculated in the SD-WACCM instead of using fixed pressures. The rest of the atmosphere is denoted as REST.

The descent of N_2_O surfaces in the polar vortex is well known^22^ and has been used to tag air-masses in the Arctic vortex to deduce chemical polar ozone loss^23,24^. Below 20 hPa, the VMRs of N_2_O were decreasing in December and January in 2005, from December to early February in 2011, from the second half of December to late January in 2006, and from early December to late January in 2009, respectively (Supplementary Figs S1–S4). These decreases in the VMRs of N_2_O indicated that a diabatic descent dominated the movements of Arctic air masses during these periods. Accordingly, the partial column of ozone (i.e., the sums of all ozone tracers) in the Arctic lower stratosphere generally showed increasing trends during these periods (Fig. 2b–e). The contours of N_2_O became flat in February 2005 and from late February to March in 2011 (Supplementary Figs S1–S2), indicating that mixing became more important. In 2006 and 2009, the major sudden stratospheric warmings occurred in late January^6,7^, which had a direct impact on the transport of stratospheric compositions over the Arctic^7,25^. Thus, in February and March, the column of Arctic lower stratospheric ozone had different tendencies during these four years (Fig. 2b–e).

For all of the studied years, the relative contributions from these source regions to Arctic lower stratospheric ozone were similar (especially in December and January), although there were some differences in the absolute values. Compared to that from the other six source regions, the ozone originating from REST had a negligible contribution (i.e., less than 10 Dobson units (DU)) to the Arctic lower stratospheric ozone in winter and spring. From December–January, the northern mid-latitudinal lower stratosphere (NMLS) was the primary source region for Arctic lower stratospheric ozone. Correspondingly, the mean absolute (percentage) contribution was 98.1 ± 6.2 DU (37.4 ± 3.7%), which is shown in Fig. 2a. Meanwhile, the tropical lower stratosphere (TPLS) and the northern mid-latitudinal middle and upper stratosphere (NMMS) were two other important source regions, which contributed 54.8 ± 1.9 DU (20.5 ± 2.5%) and 44.1 ± 14.4 DU (16.3 ± 3.6%) of the Arctic lower stratospheric ozone, respectively. The tropical middle and upper stratosphere (TPMS) only contributed 3.3 ± 1.7% of the ozone in the Arctic lower stratosphere from December–January. The local contribution (i.e., the contribution from the Arctic lower stratosphere (ARCLS)) was comparable to that from the Arctic middle and upper stratosphere (ARCMS). Each of the two regions provided approximately 10% of the Arctic lower stratospheric ozone from December–January. In February and March, the NMLS, TPLS and NMMS remained the three most important source regions for Arctic lower stratospheric ozone. Their contributions were 91.9 ± 11.2 DU (28.4 ± 1.7%), 85.9 ± 23.5 DU (26.0 ± 4.6%), and 71.9 ± 11.4 DU (22.3 ± 3.3%), respectively. Additionally, the TPMS supplied 8.6 ± 2.6% of the Arctic lower stratospheric ozone. In general, the northern mid-latitudinal components (50.2 ± 9.9%) of the Arctic lower stratospheric ozone were greater than the tropical components (29.1 ± 8.0%) in December–March from 2005–2011. These results were consistent with those of Grewe^9^.

**Figure 3:**
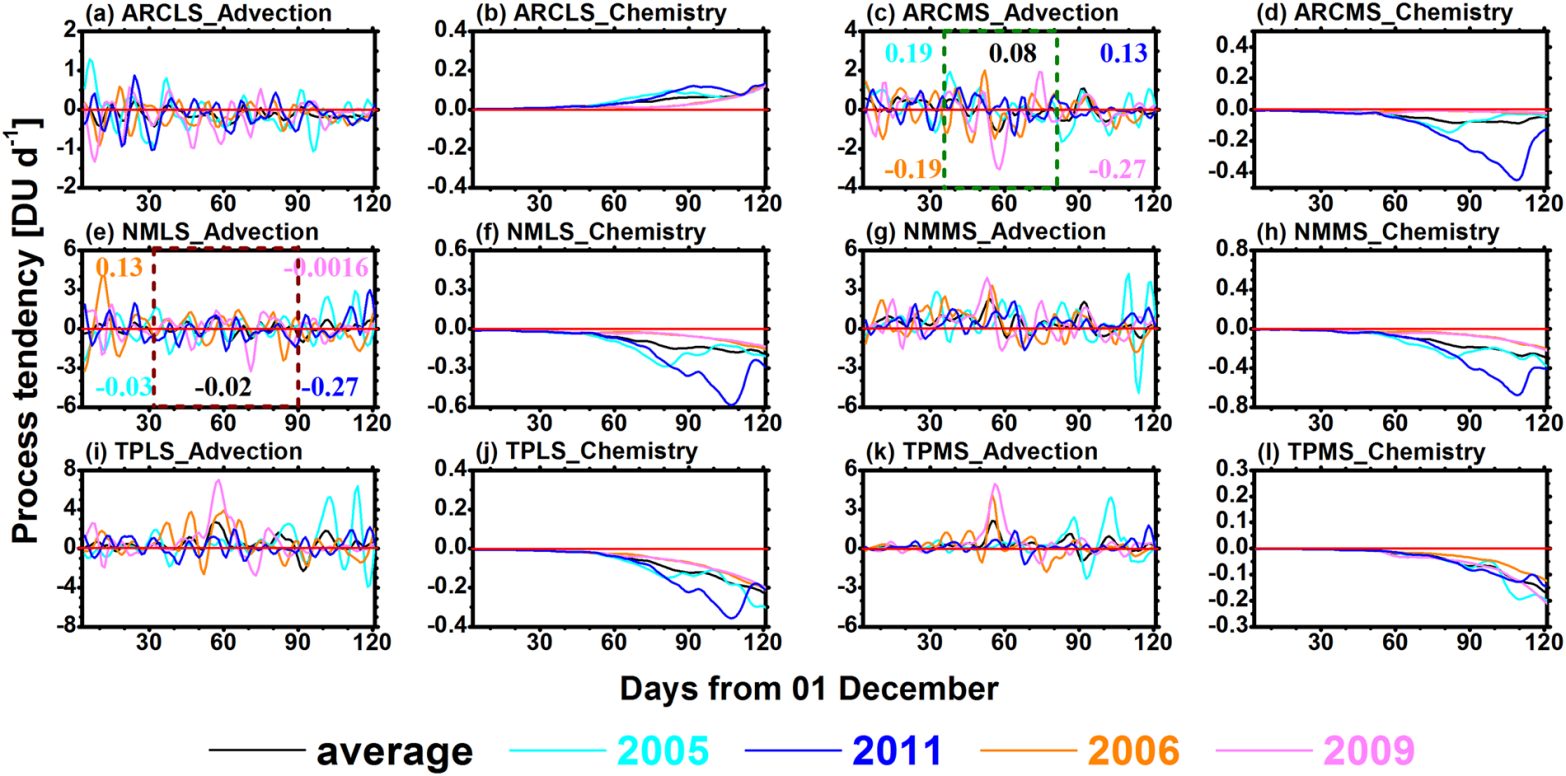
Time series of advective and net chemical tendencies (unit: DU d^−1^) for ozone tracers originating from the following regions across the Arctic: (**a**,**b**) ARCLS, (**c**,**d**) ARCMS, (**e**,**f**) NMLS, (**g**,**h**) NMMS, (**i**,**j**) TPLS, and (**k**,**l**) TPMS. All values are vertically integrated results from the tropopause to 20 hPa. The black, light blue, blue, orange, and pink lines represent the 7-year average, 2005, 2011, 2006, and 2009 results, respectively. The horizontal red line is the zero line. In Fig. 3c, the data from 05 January to 18 February are marked with a dark-green dashed box, which indicates when the mean advective tendencies during cold (warm) winters were above average (below average). Similarly, in Fig. 3e, data from 01 January to 28 February are marked with a dark-red dashed box, which indicates when the mean advective tendencies during cold (warm) winters were below average (above average). The black, light blue, blue, orange, and pink numbers in Fig. 3c,e represent the mean advective tendencies (unit: DU d^−1^) during the periods within dashed boxes for the 7-year average, 2005, 2011, 2006, and 2009 results, respectively.

In the following subsections, the contributions from various latitudinal zones to Arctic lower stratospheric ozone are analysed and discussed in detailed. To explain the temporal evolution of each latitudinal component for Arctic lower stratospheric ozone, a process analysis was used. The daily advective and net chemical tendencies of ozone tracers across the Arctic are shown in Fig. 3. Note that positive (negative) advective tendencies indicate net inflow (outflow) of ozone tracers, and positive (negative) chemical tendencies imply net chemical production (loss) of ozone tracers. Compared with the advective and chemical tendencies of ozone tracers, their tendencies for vertical diffusion were negligible in the ARCLS (Supplementary Fig. S5). Thus, the sums of the advective and net chemical tendencies of ozone tracers (Supplementary Fig. S6) were similar to the total tendencies of ozone tracers. For all of the studied years, the chemical tendencies of six ozone tracers were generally small relative to their advective tendencies from December–February, indicating that advection generally dominated contributions to Arctic lower stratospheric ozone from the six concerned sources in winter. This result was consistent with that in previous studies^1,26,27^. Additionally, because the dynamic conditions in the Arctic stratosphere were changeable in winter and spring, there is large variability in the advective tendencies of ozone tracers. In contrast, the corresponding chemical tendencies were close to zero and exhibited indistinctive variations from December–January because the chemical processes of ozone are related to solar radiation and polar nights occur in most areas of the Arctic during early winter. Artic areas experiencing sunshine conditions are expanding, and stratospheric low-temperature-induced and heterogeneous chlorine activations on polar stratospheric clouds occur; both of these conditions complicate the chemical tendencies of ozone tracers from February–March.

### The extratropical components of Arctic lower stratospheric ozone

For all of the studied years, the chemical tendencies of ARCLS ozone tracers were positive (Fig. 3b), which indicated the net chemical production of ARCLS ozone tracers. From December–February, advective tendencies were generally negative, i.e., net outflows (Fig. 3a), leading to decreases in these local contributions (Fig. 2a–e). In March, positive chemical tendencies were comparable to negative advective tendencies; thus, the decreases in the amount of ARCLS ozone tracers slowed and even became increases. In the four specific years, the summed tendencies of ARCLS ozone tracers frequently fluctuated around the 7-year average (Supplementary Fig. S6). Correspondingly, local contributions during these four years were not significantly different from the average from December–March (Fig. 2f–i). Figure 3c shows that the advective tendencies of ARCMS ozone tracers in 2005 (0.19 DU d^−1^) and those in 2011 (0.13 DU d^–1^) were both generally greater than the average (−0.08 DU d^−1^) from early January to late February. In contrast, the respective tendencies in 2006 (−0.19 DU d^−1^) and 2009 (−0.27 DU d^−1^) were both generally smaller than the average. Accordingly, there were more (less) ARCMS ozone tracers during the two cold (warm) years (Fig. 2f–i).

For all of the studied years, the advective tendencies of NMLS ozone tracers fluctuated frequently (Fig. 3e). From January–February, the mean advective tendencies in 2005 (−0.03 DU d^−1^) and 2011 (−0.27 DU d^–1^) were both below average (where the average was −0.02 DU d^−1^), which resulted in the amount of NMLS ozone tracers in 2005 (87.5 ± 5.5 DU) and 2011 (87.4 ± 5.6 DU) were both less than the 7-year average (96.3 ± 8.7 DU) (Fig. 2f,g). In contrast, the mean advective tendencies in 2006 (0.13 DU d^−1^) and 2009 (−0.0016 DU d^−1^) were generally above average (Fig. 3e), which led to larger amount of NMLS ozone tracers (107.2 ± 5.1 DU in 2006 and 96.5 ± 4.0 DU in 2009) during this period (Fig. 2h,i). In March, the inflow of NMLS ozone tracers in 2005 and 2011 increased significantly, while the advective tendencies of NMLS ozone tracers in 2006 and 2009 were both generally below average. Thus, the differences in the amount of NMLS ozone tracers between the specific years and the average result gradually decreased (Fig. 2f–i). Particularly for 2005, the sign of the anomaly became positive instead of negative in late March.

From December to early March, the advective tendencies of NMMS ozone tracers in 2011 were generally below average (Fig. 3g), which caused the amount of NMMS ozone tracers in 2011 to be smaller than the average (Fig. 2g). Then, because the advective or summed tendency became greater than the average (Fig. 3g and Supplementary Fig. S6d), the difference in the amount of NMMS ozone tracers between 2011 and the average decreased (Fig. 2g). For the other three specific years, the advective or summed tendencies fluctuated frequently around the average in winter and spring. The amount of NMMS ozone tracers during these three years had small differences from the average.

In summary, among the four extratropical source regions, the NMLS and NMMS were always two important sources for Arctic lower stratospheric ozone in winter and spring from 2005–2011. The northern mid-latitudinal stratosphere steadily contributed approximately half of the Arctic lower stratospheric ozone in winter and spring. In contrast, the percentage contributions from the Arctic stratosphere were generally less than 20%. The most visible differences between cold and warm years in terms of contributions from the northern mid-latitudes to Arctic lower stratospheric ozone generally occurred in February (Fig. 2f–i). The absolute contributions in February were smaller in cold years (151.3 ± 7.0 DU in 2005 and 139.0 ± 7.4 DU in 2011) and larger in warm years (182.6 ± 7.3 DU in 2006 and 164.6 ± 7.4 DU in 2009). However, their corresponding percentage contributions did not show evident differences between cold and warm years, with values of ~50%.

### The tropical components of Arctic lower stratospheric ozone

In 2005 and 2011, the advective tendencies of TPLS ozone tracer fluctuated around zero from December to early February (Fig. 3i), indicating no significant change in the contributions to Arctic lower stratospheric ozone from the TPLS (Fig. 2b,c). In 2006 and 2009, the inflow of TPLS ozone tracers into the Arctic increased evidently from January to early February. Thus, there were significant increases in the contributions from TPLS to Arctic ozone during the two warm years (Fig. 2d,e). After late February in 2005, the inflow of ozone from the TPLS to the Arctic increased markedly. Correspondingly, the concentration of TPLS ozone tracers increased evidently in the ARCLS. However, for the other three years, the advective tendencies did not experience similar changes, which resulted in no significant changes in the amount of TPLS ozone tracers after late February.

For all of the studied years, the advective tendencies of TPMS ozone tracers were generally small and positive for the majority of December (Fig. 3k). Therefore, the contribution from TPMS to Arctic lower stratospheric ozone showed slow and increasing trends (Fig. 2). Similar to the TPLS ozone tracers, the inflow of ozone from the TPMS to the ARCLS experienced significant increases during January and early February in the warm years. From late February to March in 2005, the inflow of TPMS ozone tracers also increased evidently. In 2011, the inflow of TPMS ozone tracers did not experience an abrupt increase from January to March.

**Figure 4:**
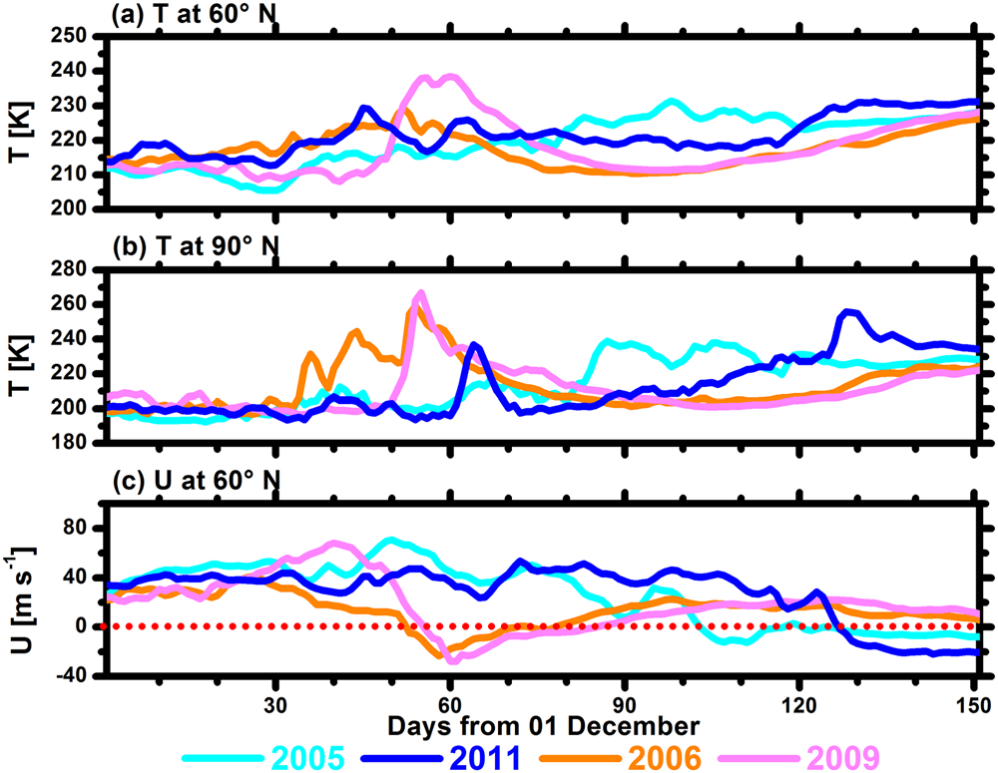
Time series of zonally averaged temperature (T; unit: K) and zonal wind (U; unit: m s^−1^) from December to April in 2005, 2011, 2006, and 2009 at 10 hPa.

According to the World Meteorological Organization, a major sudden stratospheric warming occurs if the latitudinal mean temperature increases abruptly poleward from the 60° latitude, with an associated circulation reversal over a short-time period at altitudes of 10 hPa or lower^6^. Figure 4 shows the time series of zonally averaged temperatures at 60° and 90° N and zonal winds at 60°N at 10 hPa during Arctic winters and springs in 2005, 2011, 2006, and 2009. In 2006 and 2009, Arctic stratospheric temperatures increased in January, which were accompanied by a decrease in the zonal wind speed at 60° N (major sudden stratospheric warmings occurred in late January). Accordingly, the inflows of tropical ozone in 2006 and 2009 increased significantly and were stronger than those in 2005 and 2011 from January to early February (Fig. 3i,k). Therefore, an interesting phenomenon occurred during February and March, where the tropical stratosphere contributed less ozone to the ARCLS during cold years (Fig. 2f,g), whereas it contributed more ozone to the Arctic during warm years (Fig. 2h,i). During these two months, the mean absolute (percentage) contributions from the TPLS to Arctic lower stratospheric ozone were 69.3 ± 19.9 DU (23.1 ± 4.5%) in 2005 and 55.6 ± 4.4 DU (21.9 ± 0.8%) in 2011, while they were 109.7 ± 5.5 DU (30.6 ± 1.0%) in 2006 and 107.3 ± 4.7 DU (30.4 ± 0.9%) in 2009. Meanwhile, the absolute (percentage) contributions from the TPMS to Arctic ozone were 21.1 ± 9.6 DU (6.9 ± 2.6%) in 2005 and 15.2 ± 2.0 DU (6.0 ± 0.8%) in 2011, while they were 30.6 ± 3.5 DU (8.5 ± 0.7%) in 2006 and 43.0 ± 1.7 DU (12.2 ± 0.4%) in 2009. Similarly, after late February in 2005, Arctic stratospheric temperature increased, and the zonal wind speed at 60° N decreased (the final stratospheric warming occurred in early March in 2005). Correspondingly, the inflow of tropical ozone in 2005 increased significantly after late February (Fig. 3i,k). All these results indicated that Arctic stratospheric warmings had a significant impact on the transport of ozone from the tropics to the ARCLS.

Regardless of whether the Arctic winters were cold or warm, the northern mid-latitudes steadily contributed ~50% of the Arctic lower stratospheric ozone. In contrast, the contributions from the tropics to the Arctic lower stratospheric ozone in cold years had more remarkable differences from those that occurred during warm years. For instance, major sudden stratospheric warmings occurred in late January in 2006 and 2009. In February, the tropical components during the cold years (66.5 ± 11.5 DU (24.3 ± 3.1%) in 2005 and 73.1 ± 4.7 DU (27.1 ± 1.0%) in 2011) were evidently less than those during the warm years (146.0 ± 9.0 DU (39.4 ± 1.9%) in 2006 and 153.7 ± 7.0 DU (43.0 ± 1.6%) in 2009). Additionally, the differences between the tropical components in February and those in January were 51.8 DU in 2006 and 77.0 DU in 2009, while the corresponding differences in the mid-latitudinal components were only 17.6 DU in 2006 and 18.1 DU in 2009. Therefore, a question is raised: why are the contributions from the tropics to the Arctic lower stratospheric ozone more sensitive to the stratospheric warmings than the contributions from mid-latitudes? Tropical ozone must pass through the mid-latitudinal stratosphere until it reaches the Arctic stratosphere. The chemical processes at mid-latitudes deplete tropical ozone and produce mid-latitudinal ozone at all times. The Arctic vortex acts as a barrier that suppresses the poleward transport of external air. The blocked tropical ozone remains at mid-latitudes and is depleted. Therefore, compared to the contributions from mid-latitudes to Arctic lower stratospheric ozone, the contributions from the tropics are more sensitive to stratospheric warmings (which are associated with a weakened vortex) over the Arctic.

## Conclusions

There were large interannual variations in temperature and ozone in the Arctic winter stratosphere from 2005–2011. The years 2005 and 2011 are two cold years with anomalously low Arctic ozone^4,5^, while 2006 and 2009 are two warm years in which long-lasting major sudden stratospheric warmings occurred^6,7^. Using the SD-WACCM model, in which the tagged ozone tracer method was implemented, we quantified the contributions from diverse latitudinal zones to Arctic lower stratospheric ozone in winter and spring. Our results showed that the northern mid-latitudinal stratosphere, including the NMLS and NMMS, was the primary source of Arctic lower stratospheric ozone in winter and spring from 2005–2011, with a mean percentage contribution of 50.2 ± 9.9%. The tropical stratosphere (especially the TPLS) was also an important source and contributed 29.1 ± 8.0% of the Arctic lower stratospheric ozone. The major sudden stratospheric warmings occurred during late January in 2006 and 2009^6,7^. Accordingly, the most significant differences between cold and warm Arctic winters when comparing the northern and tropical components occurred in February. The northern mid-latitudinal components in February were smaller in cold years (151.3 ± 7.0 DU in 2005 and 139.0 ± 7.4 DU in 2011) and greater in warm years (182.6 ± 7.3 DU in 2006 and 164.6 ± 7.4 DU in 2009). In contrast, the contributions from the tropical stratosphere to Arctic lower stratospheric ozone had more remarkable differences between cold and warm Arctic winters. In February, the tropical components were 66.5 ± 11.5 DU (24.3 ± 3.1%) in 2005 and 73.1 ± 4.7 DU (27.1 ± 1.0%) in 2011, while they were 146.0 ± 9.0 DU (39.4 ± 1.9%) in 2006 and 153.7 ± 7.0 DU (43.0 ± 1.6%) in 2009. Additionally, the final stratospheric warming in 2005 (early March) occurred earlier than that in 2011 (early April). Accordingly, the mean contribution from the tropics to Arctic lower stratospheric ozone in March in 2005 (112.0 DU) was also evidently greater than that in 2011 (68.6 DU). Our results also showed that for the two warm years, the differences between the tropical components in February and those in January (51.8 DU in 2006 and 77.0 DU in 2009) were significantly larger than the corresponding differences in the mid-latitudinal components (17.6 DU in 2006 and 18.1 DU in 2009). These results indicated that before and after stratospheric warmings, variations in tropical components were evidently larger than those in mid-latitudinal components. With a more detailed source apportionment than that presented in Grewe^9^, our study indicated that although the mid-latitudinal components of the Arctic lower stratospheric ozone were larger, the tropical components controlled anomalous Arctic lower stratospheric ozone in winter and spring.

## Methods

### Satellite data

The Earth Observing System MLS has been used since August 2004 to retroactively provide daily measurements of various stratospheric chemical constituents, with an 82°S–82°N horizontal coverage and a vertical resolution of 3–10 km. The MLS data are effective for studying stratospheric ozone^28^. Version 4.2 level 2 ozone data were used in this work. Before using these data, the MLS ozone product was screened based on the recommended rules in Livesey *et al*.^15^. The precision of the individual profiles of these data in the stratosphere was on the order of 0.02–0.2 ppmv^15^.

### Model and tagged ozone tracer method

The WACCM model is based on the physical parameterizations used in the Community Atmosphere Model version 4 (CAM4)^29^. The basic chemistry mechanism in the WACCM is taken from the Model for Ozone and Related Chemical Tracers version 3 (MOZART-3)^30^. In this study, the polar stratospheric cloud module from Wegner *et al*.^31^ was used in the WACCM instead of the standard module from Kinnison *et al*.^30^, which improves the performance of WACCM when simulating ozone and its related constituents^20^. The SD-WACCM, whose meteorological parameters are driven by Modern Era Retrospective-analysis for Research and Applications version 2 (MERRA-2) data^32^, was used in this study. The SD-WACCM has a horizontal resolution of 1.9° × 2.5° (latitude × longitude), with 88 levels in the vertical dimension that cover an altitudinal range from the surface to approximately 140 km. In this model, a nudging approach was used to calculate the meteorological fields^18^. Data for the horizontal winds, temperature, and surface pressure from MERRA-2 were used to drive the physical parameterization from the surface to 50 km^19^, which allowed for more accurate comparisons between the measurements of atmospheric composition and the model output^18^. Above 60 km, the simulated meteorological fields were calculated online, with a linear transition between 50 and 60 km^19^. The MERRA-2 datasets used in this study had the same resolution as that of the SD-WACCM, which are available on the Earth System Grid (https://www.earthsystemgrid.org/home.html) and are generated from the original resolution (1/2° × 2/3°) using a conservative re-gridding procedure^18,33^.

To investigate the contributions from diverse source regions to Arctic lower stratospheric ozone, we used a tagged tracer method that has been widely used in modelling studies^9,11,12,34^. In this tagged method, a tracer is tagged in a certain region, where it is emitted or chemically produced. The tagged tracer undergoes a series of atmospheric processes that the regular chemical species experiences. The temporal evolution in the concentration of an ozone tracer is determined by advective transport, chemical transformation, vertical diffusion, and surface deposition. Note that the ozone tracers provide no feedback regarding the dynamic and thermal fields and are separated from the regular ozone in the SD-WACCM. In this study, the stratosphere over the tropics and the Northern Hemisphere was divided into six source regions (Table 1). These regions were chosen so that the main ozone production area (i.e., the tropical mid-stratosphere) could be separated from extra-tropical regions in the Northern Hemisphere^9^. The rest of the atmosphere was regarded as the seventh region and denoted as REST. Different from the source regions in the work of Grewe^9^, we subdivided the Northern Hemisphere into mid- and high-latitudes to investigate their individual ozone contributions during winter and spring. Meanwhile, the tropopause was determined by the dynamic calculation in the SD-WACCM, which was ~230–360 hPa in the northern high-latitudes from December to April (rather than a fixed pressure level). The chemistry preprocessor in the WACCM can register these ozone tracers as advective species, and their temporal evolutions in advection and vertical diffusion are treated in the same manner as other constituents. The chemical tendency for ozone tracers produced in region $i$ is expressed as:

1 $$\frac{d[{O}_{3}^{i}]}{dt}={({O}_{3}^{i})}^{{\rm{prod}}}-{({O}_{3})}^{{\rm{loss}}}\frac{[{O}_{3}^{i}]}{[{O}_{3}]}$$

where $[{O}_{3}^{i}]$ represents the concentration of ozone tracers assigned to region $i$, $[{O}_{3}]$ represents the concentration of ozone, ${({O}_{3})}^{{\rm{loss}}}$ represents the ozone chemical loss, and ${({O}_{3}^{i})}^{{\rm{prod}}}$ represents the chemical production of ozone tracers assigned to region $i$. Within region $i$, ${({O}_{3}^{i})}^{{\rm{prod}}}$ is equal to the ozone chemical production; outside of region $i$, ${({O}_{3}^{i})}^{{\rm{prod}}}=0$. The chemical production and destruction of the odd oxygen family (O_x_) are used instead of O_3_ due to the nonlinearity of the O_3_ photochemistry^23^. We define O_x_ as:

2 $${{\rm{O}}}_{{\rm{x}}}={{\rm{O}}}_{3}+{\rm{O}}+{{\rm{O}}}^{1}{\rm{D}}+{\rm{ClO}}+{\rm{HOCl}}+2{{\rm{Cl}}}_{2}{{\rm{O}}}_{2}+{\rm{OClO}}+{{\rm{NO}}}_{2}+{{\rm{HNO}}}_{3}+2{{\rm{NO}}}_{3}\,+3{{\rm{N}}}_{2}{{\rm{O}}}_{5}+{{\rm{HNO}}}_{4}+2{{\rm{ClNO}}}_{3}+{{\rm{B}}}_{{\rm{r}}}{\rm{O}}+{{\rm{HOB}}}_{{\rm{r}}}+2{{\rm{B}}}_{{\rm{r}}}{{\rm{NO}}}_{3}$$

Our definition is similar to that of Grewe (http://www.pa.op.dlr.de/~VolkerGrewe/EMAC_VG.htm#ProdLoss), but without PAN, NACA, MPAN, IC_3_H_7_NO_3_, LC_4_H_9_NO_3_, and ISON, since these species are not included in the WACCM and they account for small portions of O_x_. Due to the long lifetime (ranging from months to a year) of lower stratospheric ozone, we initiated the SD-WACCM simulations on 01 January 2002 to obtain stable initial values of the ozone tracers and investigated the simulation results from 01 December to 30 April from 2004/2005–2010/2011 in this study.

### Data Availability

The datasets generated and/or analysed during the current study are available from the corresponding author upon reasonable request.

## Electronic supplementary material

Supplementary information

## References

[1] Müller R (2008). Simple measures of ozone depletion in the polar stratosphere. Atmos. Chem. Phys..

[2] World Meteorological Organization (WMO). Scientific Assessment of Ozone Depletion: 2014, Global Ozone Research and Monitoring Project–Report No. 55. Geneva, Switzerland (2014).

[3] Salawitch RJ, Wofsy SC, McElroy MB (1988). Influence of polar stratospheric clouds on the depletion of Antarctic ozone. Geophys. Res. Lett..

[4] Manney GL (2006). EOS MLS observations of ozone loss in the 2004–2005 Arctic winter. Geophys. Res. Lett..

[5] Manney GL (2011). Unprecedented Arctic ozone loss in 2011. Nature.

[6] Kuttippurath J, Nikulin G (2012). A comparative study of the major sudden stratospheric warmings in the Arctic winters 2003/2004–2009/2010. Atmos. Chem. Phys..

[7] Manney GL (2009). Aura Microwave Limb Sounder observations of dynamics and transport during the record-breaking 2009 Arctic stratospheric major warming. Geophys. Res. Lett..

[8] Seinfeld, J. H. & Pandis, S. N. *Atmospheric chemistry and physics: from air pollution to climate change* (2nd ed.) 145–146 (John Wiley & Sons, Inc., Hoboken, New Jersey, 2006).

[9] Grewe V (2006). The origin of ozone. Atmos. Chem. Phys..

[10] World Meteorological Organization (WMO). Scientific Assessment of ozone depletion: 1998, Global Ozone Research and Monitoring Project–Report No. 44. Geneva, Switzerland (1999).

[11] Sudo K, Akimoto H (2007). Global source attribution of tropospheric ozone: Long-range transport from various source regions. J. Geophys. Res..

[12] Wang Y, Logan JA, Jacob DJ (1998). Global simulation of tropospheric O_3_-NO_x_-hydrocarbon chemistry: 2. Model evaluation and global ozone budget. J. Geophys. Res..

[13] Harris NRP, Lehmann R, Rex M, von der Gathen P (2010). A closer look at Arctic ozone loss and polar stratospheric clouds. Atmos. Chem. Phys..

[14] Kuttippurath J, Godin-Beekmann S, Lefèvre F, Goutail F (2010). Spatial, temporal, and vertical variability of polar stratospheric ozone loss in the Arctic winters 2004/2005–2009/2010. Atmos. Chem. Phys..

[15] Livesey, N. J. *et al*. Earth Observing System (EOS) Aura Microwave Limb Sounder (MLS) Version 4.2x Level 2 data quality and description document. JPL D-33509 Rev. A, Jet Propulsion Laboratory, California Institute of Technology, Pasadena, California (2015).

[16] Marsh DR (2013). Climate change from 1850 to 2005 simulated in CESM1 (WACCM). J. Climate.

[17] Newman PA, Gleason JF, McPeters RD, Stolarski R (1997). Anomalously low ozone over the Arctic. Geophys. Res. Lett..

[18] Lamarque J-F (2012). CAM-chem: description and evaluation of interactive atmospheric chemistry in the Community Earth System Model. Geosci. Model Dev..

[19] Kunz A, Pan LL, Konopka P, Kinnison DE, Tilmes S (2011). Chemical and dynamical discontinuity at the extratropical tropopause based on START08 and WACCM analyses. J. Geophys. Res..

[20] Brakebusch M (2013). Evaluation of Whole Atmosphere Community Climate Model simulations of ozone during Arctic winter 2004–2005. J. Geophys. Res.-Atmos..

[21] Solomon S, Kinnison D, Bandoro J, Garcia R (2015). Simulation of polar ozone depletion: An update. J. Geophys. Res.-Atmos..

[22] Bauer R (1994). Monitoring the vertical structure of the Arctic polar vortex over northern Scandinavia during EASOE: Regular N_2_O profile observations. Geophys. Res. Lett..

[23] Harris NRP (2002). Comparison of empirically derived ozone loss rates in the Arctic vortex. J. Geophys. Res..

[24] Tilmes S, Müller R, Grooß J-U, Russell JM (2004). Ozone loss and chlorine activation in the Arctic winters 1991–2003 derived with the tracer-tracer correlations. Atmos. Chem. Phys..

[25] Tao M (2015). Impact of the 2009 major stratospheric sudden warming on the composition of the stratosphere. Atmos. Chem. Phys..

[26] Chipperfield MP, Jones RL (1999). Relative influences of atmospheric chemistry and transport on Arctic ozone trends. Nature.

[27] Tegtmeier S, Rex M, Wohltmann I, Krüger K (2008). Relative importance of dynamical and chemical contributions to Arctic wintertime ozone. Geophys. Res. Lett..

[28] Froidevaux L (2008). Validation of Aura Microwave Limb Sounder stratospheric ozone measurements. J. Geophys. Res..

[29] Neale RB (2013). The Mean Climate of the Community Atmosphere Model (CAM4) in Forced SST and Fully Coupled Experiments. J. Climate.

[30] Kinnison DE (2007). Sensitivity of chemical tracers to meteorological parameters in the MOZART-3 chemical transport model. J. Geophys. Res..

[31] Wegner T, Kinnison DE, Garcia RR, Solomon S (2013). Simulation of polar stratospheric clouds in the specified dynamics version of the whole atmosphere community climate model. J. Geophys. Res.-Atmos..

[32] Gelaro R (2017). The modern-era retrospective analysis for research and applications, version 2 (MERRA-2). J. Climate.

[33] Pan, C., Zhu, B., Gao, J., Kang, H. & Zhu, T. Quantitative identification of moisture sources over the Tibetan Plateau and the relationship between thermal forcing and moisture transport. *Clim. Dyn.*, 10.1007/s00382-018-4130-6 (2018).

[34] Li, Q. *et al*. Transatlantic transport of pollution and its effects on surface ozone in Europe and North America. *J. Geophys. Res*. **107** (2002).

