# Supplementary material for: Quantifying Arctic lower stratospheric ozone sources in winter and spring
Source: Sci Rep. 2018 Jun 12;8:8934. doi: 10.1038/s41598-018-27045-5 (PMC5997751; doi:10.1038/s41598-018-27045-5)
Supplement: Supplementary file 1 — Supplementary information [file 41598_2018_27045_MOESM1_ESM.pdf]

Supplementary information of "Quantifying Arctic lower stratospheric ozone sources in winter and spring"

Chen Pan[1,2,3,4], Bin Zhu[*1,2,3,4], Jinhui Gao[1,2,3,4], Xuewei Hou[1,2,3,4], Hanqing Kang[1,2,3,4], Dongdong Wang[1,2,3,4]

[1]Key Laboratory for Aerosol-Cloud-Precipitation of China Meteorological Administration, Nanjing University of Information Science & Technology, Nanjing, China; [2]Collaborative Innovation Center on Forecast and Evaluation of Meteorological Disasters, Nanjing University of Information Science & Technology, Nanjing, China; [3]Key Laboratory of Meteorological Disaster, Ministry of Education (KLME), Nanjing University of Information Science & Technology, Nanjing, China; [4]Joint International Research Laboratory of Climate and Environment Change (ILCEC), Nanjing University of Information Science & Technology, Nanjing, China

Corresponding author: Bin Zhu (binzhu@nuist.edu.cn)

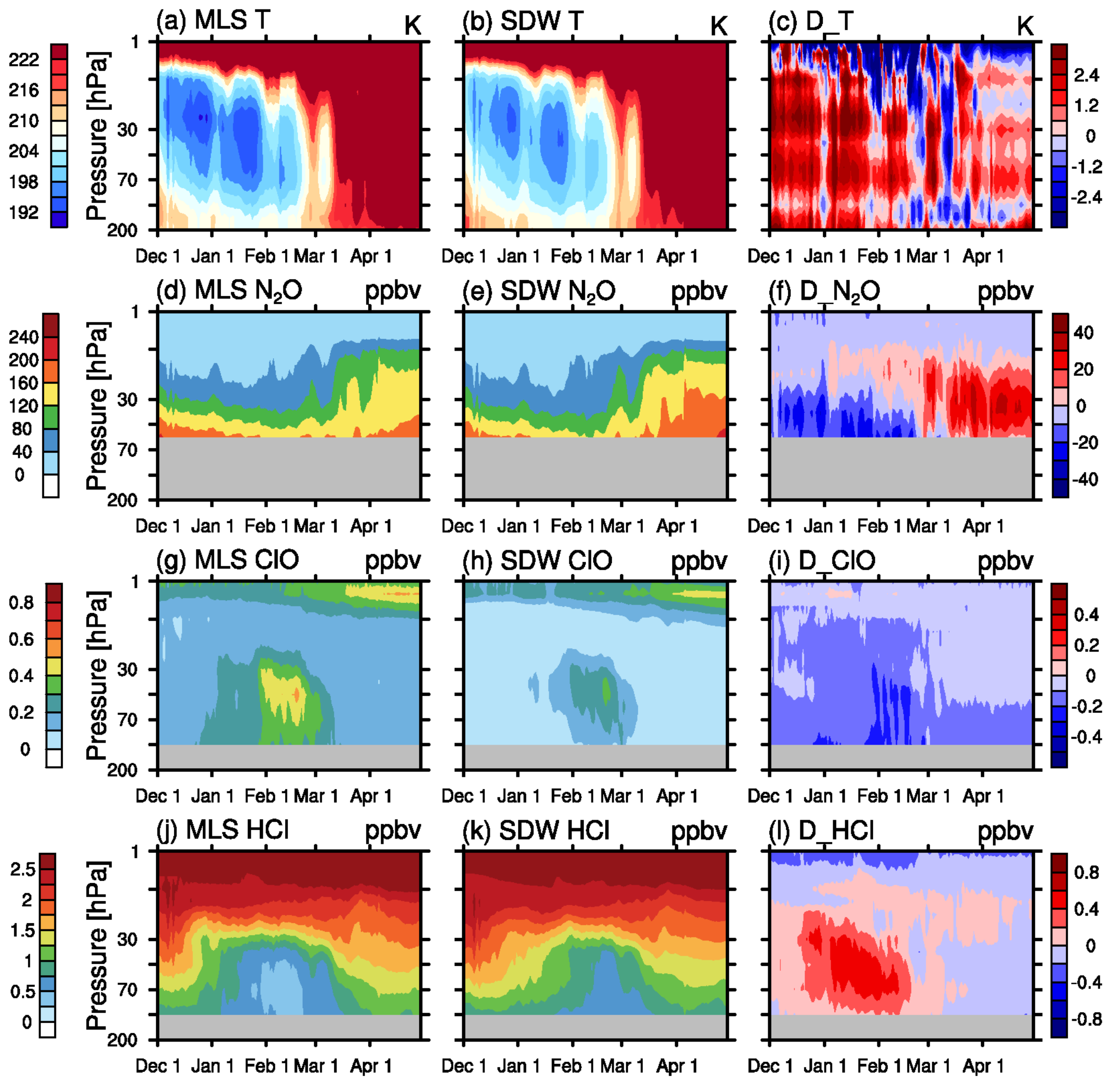

Supplementary Figure S1. Evolutions of daily (left) MLS observations, (center) SD-WACCM simulations, and (right) their differences in temperature, N_2O, ClO, and HCl over the Arctic from December to April of 2005. The grey areas indicate where data were either missed or acquired outside of the Arctic.

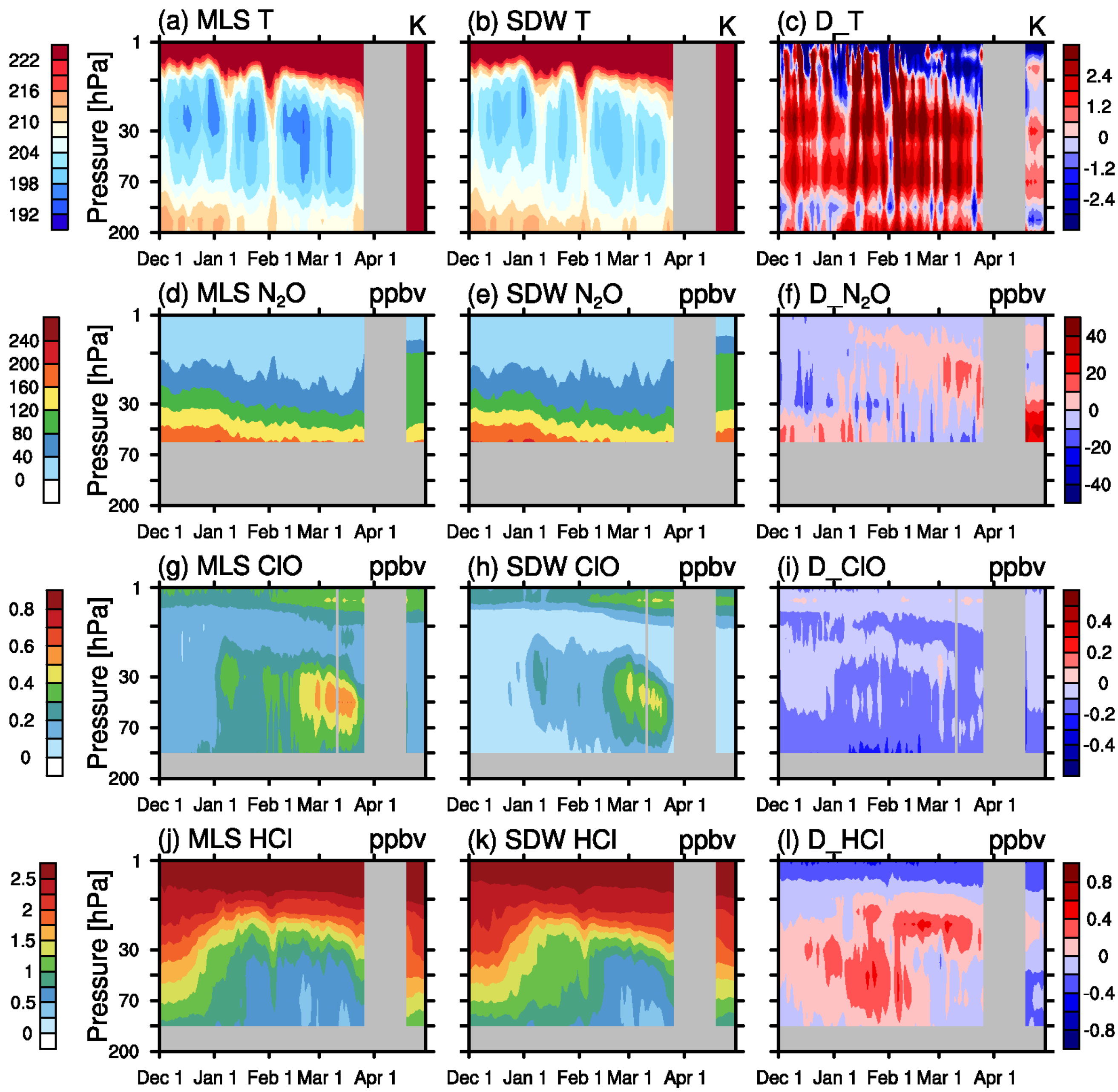

Supplementary Figure S2. Evolutions of daily (left) MLS observations, (center) SD-WACCM simulations, and (right) their differences in temperature, N_2O, ClO, and HCl over the Arctic from December to April of 2011. The grey areas indicate where data were either missed or acquired outside of the Arctic.

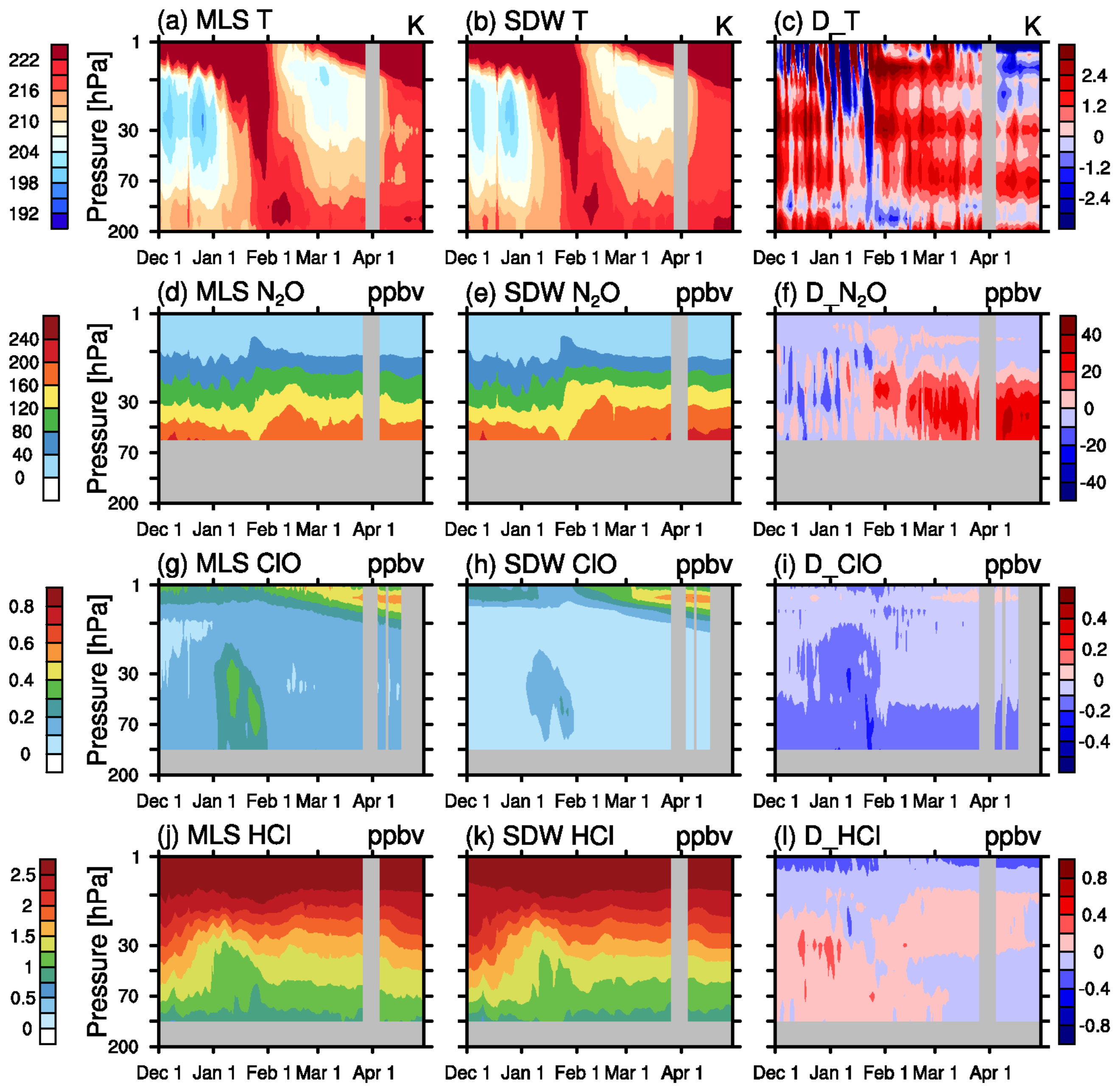

Supplementary Figure S3. Evolutions of daily (left) MLS observations, (center) SD-WACCM simulations, and (right) their differences in temperature, N_2O, ClO, and HCl over the Arctic from December to April of 2006. The grey areas indicate where data were either missed or acquired outside of the Arctic.

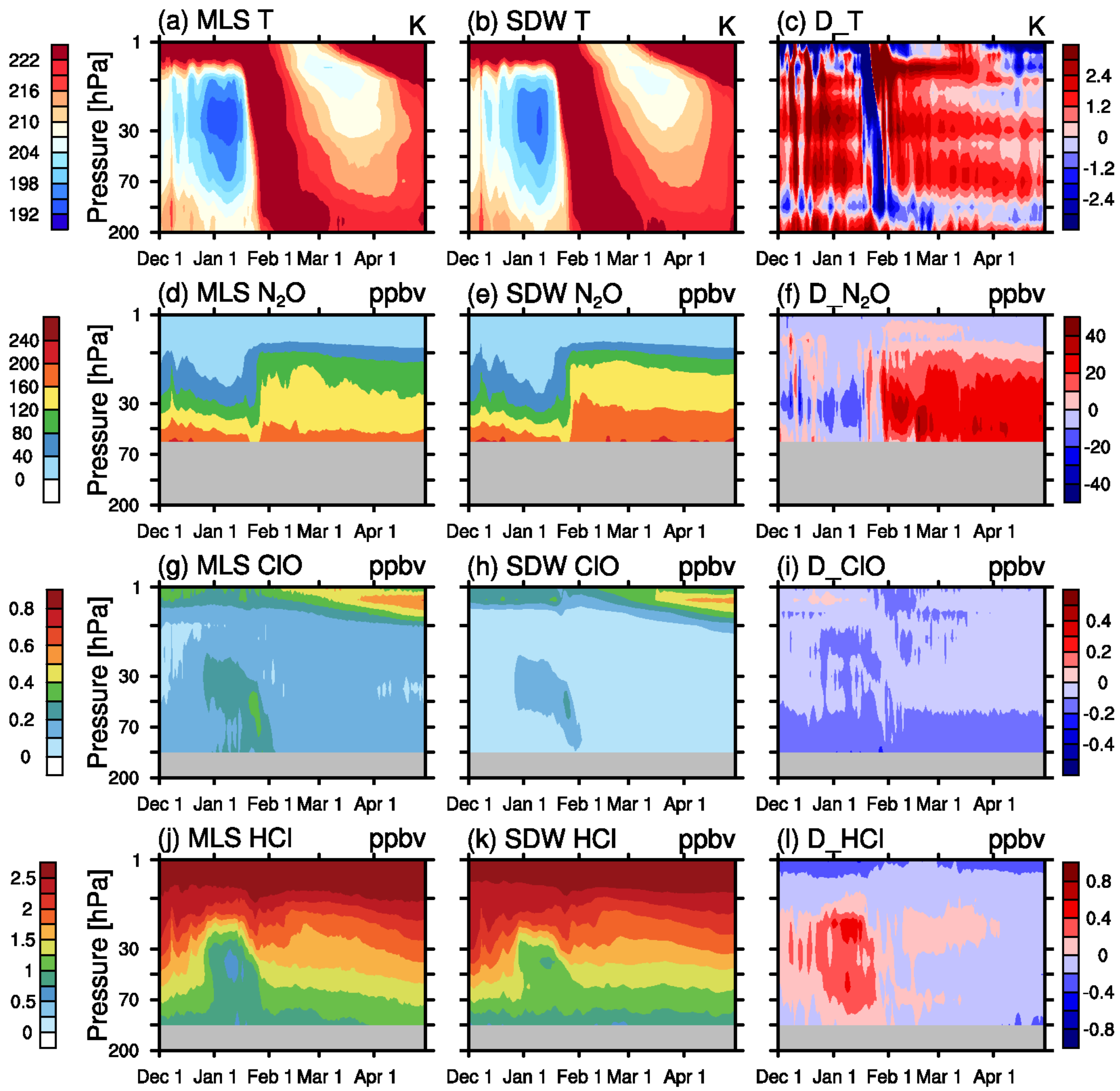

Supplementary Figure S4. Evolutions of daily (left) MLS observations, (center) SD-WACCM simulations, and (right) their differences in temperature, N_2O, ClO, and HCl over the Arctic from December to April of 2009. The grey areas indicate where data were either missed or acquired outside of the Arctic.

The top rows of Supplementary Figs. S1–S4 show the comparisons between the MLS observed and SD-WACCM simulated Arctic temperatures between 200 and 1 hPa from December to April of 2005, 2011, 2006, and 2009, respectively. The overall evolutions of simulated temperatures were comparable to the MLS measurements in the four years. Supplementary Figs. S1c, S2c, S3c, and S4c show that the simulated temperatures were generally 0.6–3 K higher than the MLS temperatures between 100 and 1 hPa, which exceeded the precision (±0.5– ±0.7 K) of MLS temperatures[1].

The long lifetime of nitrous oxide (N_2O) in the stratosphere makes N_2O a suitable tracer to study transport process in the lower stratosphere[2]. Panels d–e of Supplementary Figs. S1–S4 show the observed and simulated distributions of N_2O across the Arctic from December to April. In the four Arctic winters, the SD-WACCM and MLS N_2O compared reasonably well. The decreasing volume mixing ratio (VMR) of N_2O below 20 hPa confirmed that subsidence dominated the movements of Arctic air masses in December and January in 2005[3], from December to early February in 2011, from the second half of December to January in 2006, and from early December to late January in 2009. The contours became flat in February in 2005 and from late February to March in 2011, indicating that mixing became more important. In 2005, the Arctic vortex broke up after early March as suggested by Manney et al.[3], with N_2O increasing rapidly. The major sudden stratospheric warmings occurred during late January in 2006 and 2009[4–5], with the zonally averaged wind reversal rapidly. Therefore, the VMRs of N_2O increased rapidly after late January in 2006 and 2009.

The comparisons between observed and simulated Arctic chlorine monoxide (ClO) are shown in the third rows of Supplementary Figs. S1–S4. In general, SD-WACCM can characterize the evolutions of MLS observed ClO fairly well for these four years. In 2005, significant increases in ClO occurred from January to early March between 100 and 20 hPa. In 2011, such increases happened from early January to March below 10 hPa. In 2006, there were evident increases in ClO during January below 10 hPa. In 2009, significant increases in ClO occurred from late December to early February below 20 hPa. There were, however, significant differences between the MLS and SD-WACCM ClO. The SD-WACCM ClO was generally 0.2–0.3 ppbv smaller than the MLS measurement below 10 hPa, which exceeded the precision (±0.1 ppbv) of MLS ClO between 100 and 10 hPa[1].

Qualitatively, SD-WACCM can characterize the overall evolutions of hydrochloric acid (HCl) reasonably well for these four years, as shown in the bottom rows of Supplementary Figs. S1–S4. In 2005, the VMR of HCl decreased from December to February and increased throughout March below 20 hPa. In 2011, the VMR of HCl decreased from December to early March and then increased below 10 hPa. In 2006 and 2009, HCl generally decreased from December to late January and then increased below 10 hPa. Supplementary Fig. S1l showed that the simulated HCl was 0.2–0.4 ppbv greater than the MLS HCl from late December to February in 2005 below 10 hPa. In this period, the decrease in VMR of HCl was caused primarily by heterogeneous chemical reactions of HCl. The overestimate of simulated HCl can lead to too little chlorine activation, and thus overestimate of ozone (Fig. 1c). Similarly, the simulated HCl was biased high relative to the MLS measurement by 0.2–0.4 ppbv from late December to early February in 2011 below 20 hPa. Additionally, the simulated HCl was 0.2–0.4 ppbv greater than MLS HCl from late January to March in 2011 between 30 and 10 hPa (Supplementary Fig. S2l). In the two warm years (2006 and 2009), the simulated HCl was generally biased high 0.2 ppbv relative to MLS HCl during December and January (Supplementary Figs. S3l and S4l). The overestimates of HCl also indicated that SD-WACCM underestimated chlorine activations in 2011, 2006, and 2009.

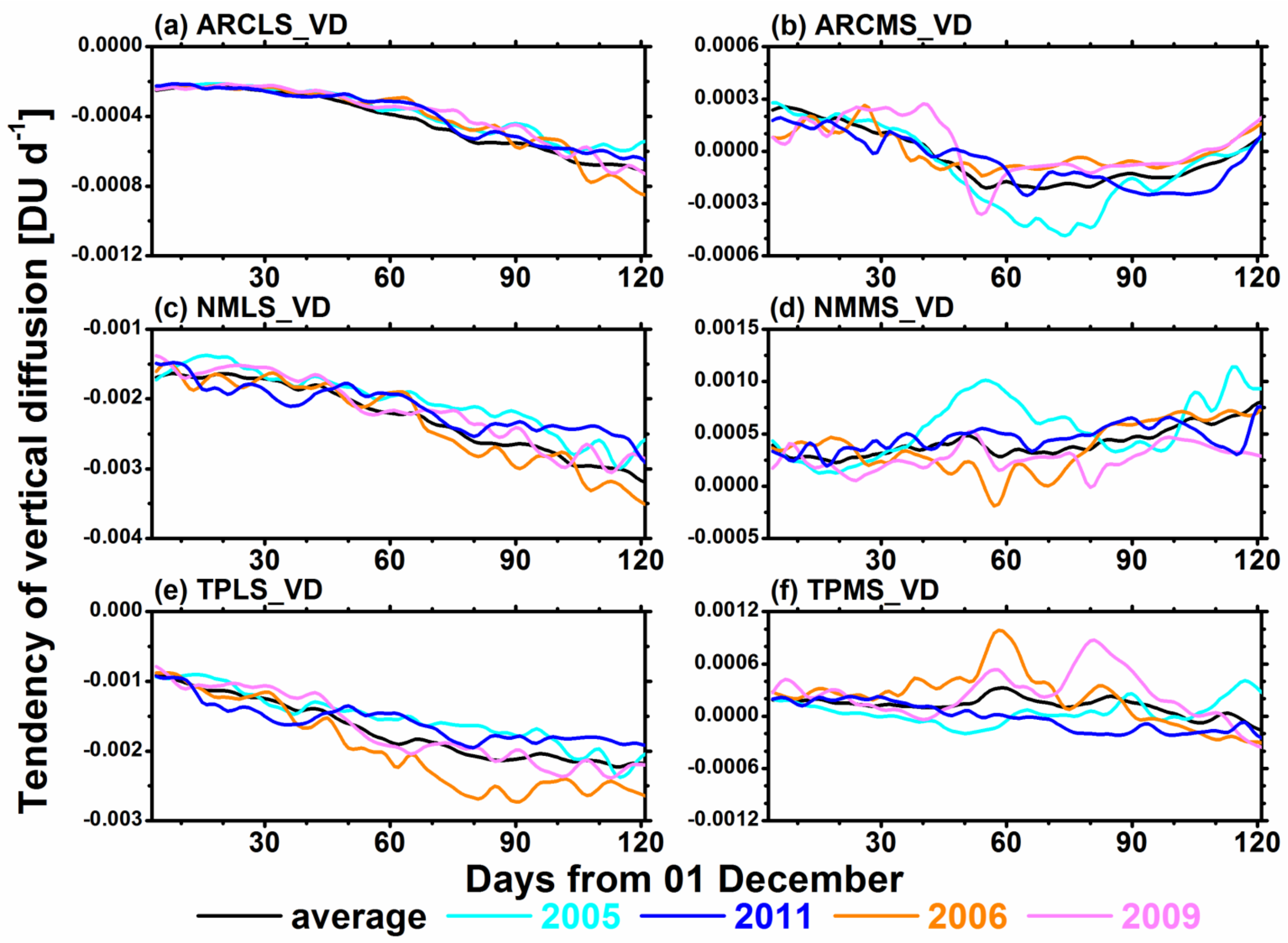

Supplementary Figure S5. Time series of the tendencies (unit: DU d^{-1}) of ozone tracers originating from the following regions across the Arctic: (a) ARCLS, (b) ARCMS, (c) NMLS, (d) NMMS, (e) TPLS, and (f) TPMS in vertical diffusion. All values are vertically integrated results from the tropopause to 20 hPa. The black, light blue, blue, orange, and pink lines represent the 7-year average, 2005, 2011, 2006, and 2009 results, respectively.

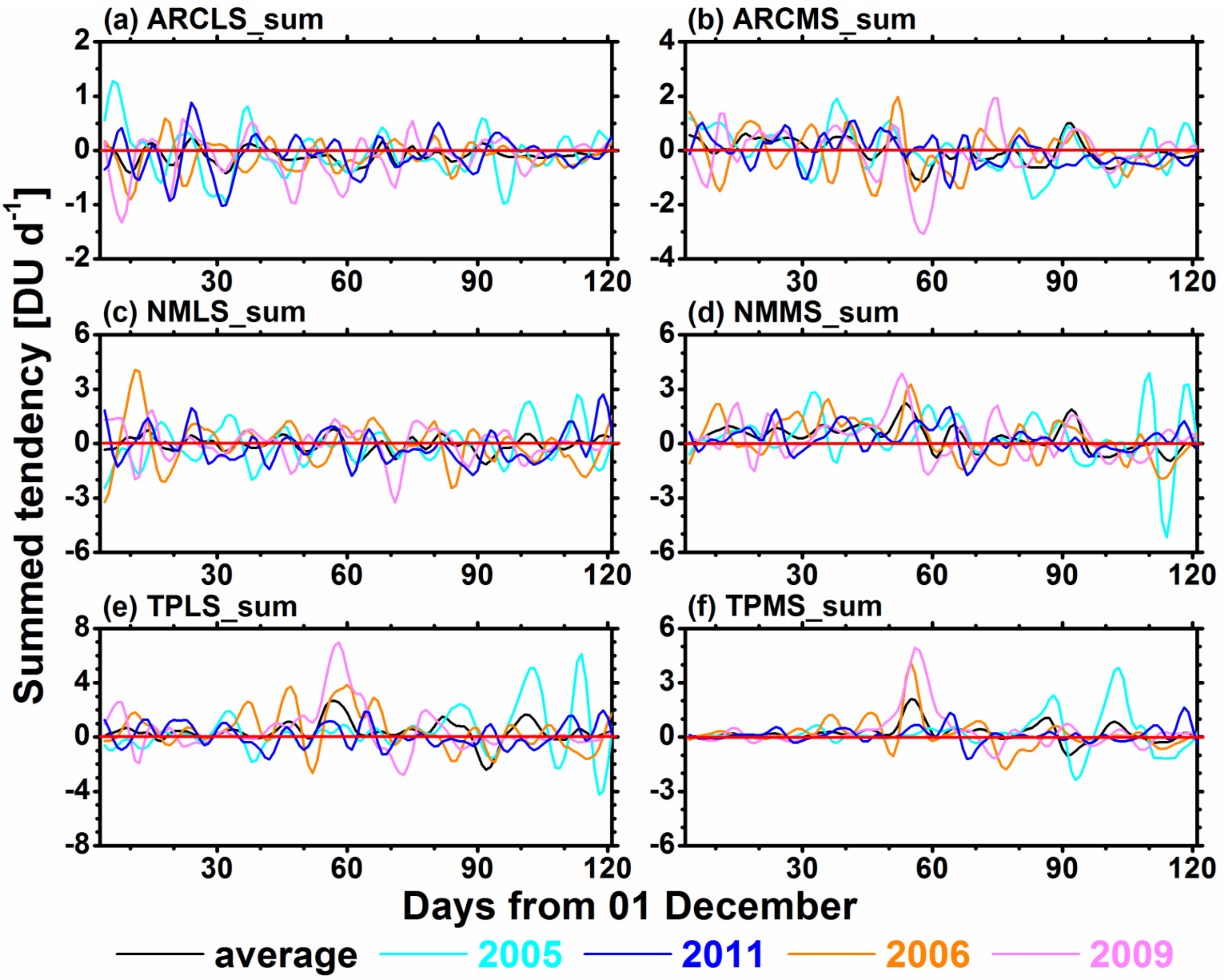

Supplementary Figure S6. Time series of the sums of advective and net chemical tendencies (unit: DU d^{-1}) for ozone tracers originating from the following regions across the Arctic: (a) ARCLS, (b) ARCMS, (c) NMLS, (d) NMMS, (e) TPLS, and (f) TPMS. All values are vertically integrated results from the tropopause to 20 hPa. The black, light blue, blue, orange, and pink lines represent the 7-year average, 2005, 2011, 2006, and 2009 results, respectively. The horizontal red line is the zero line.

References

1. Livesey, N. J. *et al*. Earth Observing System (EOS) Aura Microwave Limb Sounder (MLS) Version 4.2x Level 2 data quality and description document. JPL D-33509 Rev. A, Jet Propulsion Laboratory, California Institute of Technology, Pasadena, California (2015).
2. Loewenstein, M., Podolske, J. R., Chan, K. R. & Strahan, S. E. N_2O as a dynamical tracer in the Arctic vortex. *Geophys. Res. Lett.* **17**, 477–480 (1990).
3. Manney, G. L. *et al*. EOS MLS observations of ozone loss in the 2004–2005 Arctic winter. *Geophys. Res. Lett.*, **33**, L04802 (2006).
4. Kuttippurath, J. & Nikulin, G. A comparative study of the major sudden stratospheric warmings in the Arctic winters 2003/2004–2009/2010. *Atmos. Chem. Phys.*, **12**, 8115–8129 (2012).
5. Manney, G. L., *et al*. Aura Microwave Limb Sounder observations of dynamics and transport during the record-breaking 2009 Arctic stratospheric major warming. *Geophys. Res. Lett.*, **36**, L12815 (2009).
